# Protocol for a Delphi consensus exercise to identify a core set of criteria for selecting health related outcome measures (HROM) to be used in primary health care

**DOI:** 10.1186/s12875-018-0831-5

**Published:** 2018-09-04

**Authors:** Pasqualina Santaguida, Lisa Dolovich, Doug Oliver, Larkin Lamarche, Anne Gilsing, Lauren E. Griffith, Julie Richardson, Dee Mangin, Monika Kastner, Parminder Raina

**Affiliations:** 1 Department of Health Research Methods, Evidence and Impact, McMaster University, HSC 3N50-G, 1280 Main Street West, Hamilton, Ontario L8S 4L7 Canada; 2 Department of Family Medicine, DBHSC, McMaster University, 5th Floor 100 Main St West, Hamilton, ON L8P 1H6 Canada; 3 Leslie Dan Faculty of Pharmacy, University of Toronto, Toronto, Ontario M5S 3M2 Canada; 4 Department of Family Medicine, DBHSC, McMaster University, 3rd Floor, 100 Main St West, Hamilton, ON L8P 1H6 Canada; 5 Department of Health Research Methods, Evidence and Impact, McMaster University, 175 Longwood Rd. S. Suite 207A, Hamilton, ON L8P 0A1 Canada; 6 Department of Health Research Methods, Evidence and Impact, McMaster University, 175 Longwood Rd. S. Suite 309A, Hamilton, ON L8P 0A1 Canada; 7 School of Rehabilitation Sciences, McMaster University, 1400 Main St. W. IAHS 443, Hamilton, ON L8S 1C7A Canada; 8 Department of Family Medicine, DBHSC, McMaster University, 5th Floor, 100 Main St West, Hamilton, Ontario L8P 1H6 Canada; 9 Institute of Health Policy, Management and Evaluation, University of Toronto, 155 College Street, Toronto, ON M5T 3M6 Canada; 10 Department of Health Research Methods, Evidence and Impact, McMaster University, 175 Longwood Rd. S. Suite 309A, Hamilton, ON L8P 0A1 Canada

**Keywords:** Delphi consensus, Selection of outcomes, Primary health care, Protocol

## Abstract

**Background:**

Promoting the collection and use of health related outcome measures (HROM) in daily practice has long been a goal for improving and assessing the effectiveness of care provided to patients. However, there has been a lack of consensus on what criteria to use to select outcomes or instruments, particularly in the context of primary health care settings where patients present with multiple concurrent health conditions and interventions are whole-health and person-focused. The purpose of this proposed study is to undertake a formal consensus exercise to establish criteria for selecting HROM (including patient-reported (PRO or PROM), observer-reported (ObsR)), clinician-reported (ClinRO) and performance related outcomes (PerfO) for use in shared decision-making, or in assessing, screening or monitoring health status in primary health care settings.

**Methods:**

A Delphi consensus online survey will be developed. Criteria for the Delphi panel participants to consider were selected from a targeted literature search. These initial criteria (*n* = 35) were grouped into four categories within which items will be presented in the Delphi survey, with the option to suggest additional items. Panel members invited to participate will include primary health care practitioners and administrators, policy-makers, researchers, and experts in HROM development; patients will be excluded. Standard Delphi methodology will be employed with an expectation of at least 3 rounds to achieve consensus (75% agreement). As the final list of criteria for selecting HROM emerges, panel members will be asked to provide opinions about potential weighting of items. The Delphi survey was approved by the Ethics Committee in the Faculty of Health Sciences at McMaster University.

**Discussion:**

Previous literature establishing criteria for selecting HROM were developed with a focus on patient reported outcomes, psychological/ behavioural outcomes or outcomes for minimum core outcome sets in clinical trials. Although helpful, these criteria may not be applicable and feasible for application in a primary health care context where patients with multi-morbidity and complex interventions are typical and the constraints of providing health services differ from those in research studies. The findings from this Delphi consensus study will address a gap for establishing consensus on criteria for selecting HROM for use across primary health care settings.

## Background

Collection of outcomes for use in evaluation of health care, quality improvement, clinical decision-making or effectiveness research continues to be promoted and encouraged. Routine collection of health related outcomes can occur in more formal contexts, for example in developing patient or disease registries, or less formal where findings are documented in individual patient charts. Collection of health related outcomes can be used at a system level or for individual patient management. In either case, the dilemma remains as to which health related outcomes to select that accurately captures the intended purposes for collection. There are a large number of criteria that could be considered to select outcomes which makes the process challenging.

Several attempts to establish a minimum or core set of criteria used to evaluate or select outcomes have been reported [1–6]. However, there are important limitations in these previous recommendations. Some focus predominately on patient reported [1] or psychological/ behavioural outcomes [2, 3]; there are many other types of measures used to assess the impact of care. Another attempt was developed in the context of comparing clinical trials and using an agreed upon set of common outcomes [4]. Similarly another is to be considered broadly at a systems level and not readily operationalized with consistency [5, 6]. Although helpful, these previous criteria recommended to select outcomes may not be applicable for all types of outcomes or in other contexts in which they were developed.

This paper will detail a protocol to establish criteria for selecting health related outcome measures (HROM) in the context of primary health care (PHC). PHC is characterized by patients with multifactorial health problems and accordingly the interventions are multilayered and complex. We propose a Delphi consensus study that includes experts in HROM development and clinical research. We provide background information around definitions and use of HROM, previous attempts to establish criteria to select measures, hypothesized benefits and use of HROM in clinical settings, the relevance to the PHC context and evaluation of complex interventions generally. The proposed research protocol details are specified and previous gaps in the literature are addressed. The overall goal of this research is to contribute to the development of a transparent and reproducible process to select HROM that will be used across different sites that are implementing and adapting complex PHC interventions.

### Purpose and definition of outcomes and health related outcome measures

Apart from the practical and logistical aspects of routine collection of health related outcomes for evaluation purposes, there are some challenges conceptually in defining what is and is not an outcome. This is related to the variation in definitions of an outcome generally, as well as, definitions of an outcome measure and an outcome assessment [7–23]. The term outcome is often used in a global sense to connect the expected impact of an intervention. It is also used frequently to refer to instruments or scales used to assess or evaluate interventions or patient status at any point in the health care trajectory. The term outcome may be confused with the term endpoint (which sometimes is defined as a group of outcomes used to establish the benefit or harm of an intervention).

For the purposes of this paper we have adopted the definition of an outcome as one that captures a “measureable characteristic that is influenced or affected by an individuals’ baseline state or an intervention as in a clinical trial or other exposure” [8, 19]. An outcome could reflect both the benefit or harm to a patient who receives an intervention and exposure [10]. This definition is in keeping with the Donabedian framework for quality improvement which defines outcome as the “effects of care on the health status of patients and populations” [11]. Outcome assessment is considered to be the most important of three categories of assessment of quality of care (including structure (context where care is delivered and including buildings, equipment, staffing, etc.) and process (interactions between patients, providers, system) [11, 24, 25]. In this quality improvement framework, the focus of outcomes is on the impact of care on the person receiving it. Ideally the assessment of outcome of care should always reflect what patients actually experience and consider important [26]. Thus HROM are measures or scales or instruments that are designed to capture outcomes (domains and constructs) in a health care context.

What is key in this definition of outcomes and HROM is that it is not the specific type of measure or instrument or test that is used in the assessment but rather the intended purpose of the outcome measurement and the timing of the collection during care receiving. For example, consider hemoglobin A1c in the care of patients with diabetes. Capture of the frequency of measurement of hemoglobin A1c can be used to assess health service processes; estimation of the mean level (or mean change) can be used to assess the impact of the health services outcome (on the recipient). The influential Food and Drug Administration (FDA) in the United States considers a *clinical* outcome *assessment* as one that may use any of four primary types of “outcome measures” that would reflect domains important to relevant stakeholders and these include: patient reported outcome measures (PROM) or (PRO), observer reported outcomes (ObsRO), clinician reported outcomes (ClinRO), and performance outcomes (PerfO). In the FDA regulatory process, laboratory tests, biomarkers, and even mortality/survival are also important types of outcomes that can be used to assess the effectiveness of interventions (in their regulatory capacity, this refers to drugs, devices and biomarkers) for which approval is sought. This broad understanding of HROM and their potential classification are useful in understanding outcome assessment generally.

### Use of HROM as an intervention

Promoting the capture of HROM in daily practice has long been a goal for assessing the effectiveness of care provided to patients [27]. However, it is only recently that there is increasing recognition that the routine collection and interpretation of HROM in practice can be shown to influence ongoing care, such that it becomes an intervention in and of itself. Greenhalgh [28, 29] hypothesized pathways of influence and impact of using PROM in clinical settings. Within this hypothesized model, the use of PROM (a type of HROM) will prompt clinicians to discuss health related quality of life concerns, it will enable clinicians to detect unrecognised problems, and will likely change the way in which a clinician will respond (as the findings from the PROM may show a change in the health related quality of life . The act of reviewing or using PROM serves to monitor treatment potentially resulting in changes to the patient’s behaviour and improvement in the patient’s overall health status or satisfaction with care. Thus, outcome assessment by collecting HROM not only evaluates the impact of an intervention at the resolution or end of a particular episode of care, but it can be considered an added “intervention” that serves to direct or modify care. For example, the rate of screening for depression is one outcome that could be used to demonstrate the impact of a routine visit to a family doctor. However, if the screening result is positive, then the physician will now direct care to address this previously undetected health problem. In the context of PHC, like other areas of health care provision, a wide spectrum of HROM are used to assess both simple and complex interventions and single and comorbid health conditions. Added to this complexity is that most visits to PHC are for symptoms or complaints rather than diseases so the range of available HROM related to single diseases are less appropriate [30].

It is not clear which of types of HROM should be selected to provide useful information to assess the impact of care or to allow clinicians and decision-makers to direct or modify care. What is an ideal HROM in one context may not be so in another context and in part reflects the intended purpose of the outcome assessment. The challenge for clinicians, researchers and decision-makers is to select the most appropriate HROM in light of the fact that likely several well established HROM currently exist and are candidates for selection. Although there have been some attempts to set some general guidelines for selecting HROM for outcome assessment, these criteria have several key limitations that have been noted and none provide recommendations for dealing with equally valid but competing measures.

### Hypothesized benefits of using HROM in clinical settings

Donabedian was one of the first to propose that structure, process and outcomes are seminal to evaluating quality of care [24, 25]. Since the late 1960s there has been great emphasis on the selection and use of HROM in quality assessment but also in effectiveness research and even directing clinical decision-making for individual patients. As noted previously, Greenhalgh [28, 29] hypothesized pathways of influence and impact of using PROM in clinical settings. Within this model, PROM can be considered as having multiple purposes that include “clinical tests”, “interventions” and “outcomes”. Table 1 details how the model specifies the clinical goal when using PROM and the potential impact of using these in clinical settings. Note that this model may be applied to HROM, as the definition of HROM includes PROM (as well as, ObsRO, ClinRO, PerfO).

**Table 1:** Greenhalgh [24] model hypothesizing pathways of influence and impact in using PROM in clinical settings

Clinical Goal of PROM	Impact of PROM use
Change the content and nature of the communication between the family doctor/ primary health care team and the patient/consumer to improve patient centered care	Changes to patient behaviour
Monitor health status or response to treatment	Changes to the clinical management of patient’s/consumers
Detect unrecognized problems or screen for disease problems in those at high risk	Improved health outcomes and satisfaction of patient
	Facilitation of communication amongst multidisciplinary teams
	Evaluation of the effectiveness of routine care and assessing the quality of care

When considering the clinical goals (i.e., assess change, monitor, detect, etc.) for using an HROM, it is not clear if the criteria used to select measures will vary as a function of the specific purpose. It is likely that the criteria may vary not only as a function of the clinical purpose for using the HROM, but also the health domain being assessed by the tool and for other administrative concerns. Soliciting expert opinions on this issue would add to the knowledge of how to apply HROM for clinical, administrative, or research purposes.

### Primary health care context and complex interventions

In the context of PHC, there is increasing recognition that most interventions are complex. The Medical Research Council (United Kingdom) has been instrumental in conceptualizing the issues related to developing, evaluating, and reporting on complex interventions [31]. Complex interventions are characterized by having a number of interactions between components and requiring a number of different behaviours by those delivering or receiving the intervention [31]. Adding to this definition is that a number of groups and organizational levels are affected by the intervention. Although some would make the distinction between the complexity of the intervention versus the complexity of the health system [32] the two components interact. Flexibility and adaptation characterize complex interventions [31]. Practically this means that changes in implementation of an intervention are to be expected. More importantly, it follows that a number of outcomes may be required, as well as variability in outcomes assessing the same attribute of interest [31]. This would suggest that adaptations of interventions in one component of a health system may result in the need to select different or modify existing HROM relative to that context. The benefit of this is that it reflects the varying and flexible aspects of PHC interventions and therefore reflects strengths of the interventions in different settings.

The development of this Delphi consensus study is considered in the context of such a complex intervention. Health TAPESTRY is a novel intervention currently being implemented in Canadian PHC settings and undergoing evaluation in a randomized trial. The intervention components involve patients, volunteers, health care teams, and researchers, as well as, the use of an electronic patient health record accessed by all relevant stakeholders [33]. There are several affiliates of Health TAPESTRY across different sites in Canada that vary in their patient populations and focus in PHC services. As expected, each Health TAPESTRY site currently uses different HROM (or will need to select new HROM), and this may present challenges for evaluating benefits and quality of this complex intervention. One overriding goal of Health TAPESTRY is to integrate new and old functionality within and across each of these centres. As the Health TAPESTRY intervention is being adapted and implemented it did not seem feasible to enforce standardization of HROM. However, stakeholders acknowledge that there is a need to guide selection of key HROM to be used in evaluating the impact of the Health TAPESTRY intervention across implementation sites. Health TAPESTRY serves as a quintessential example of PHC issues and the complexity of evaluation. Recognizing the potential for differences across Health TAPESTRY sites, a valid and trustworthy process to review and accept suggestions for HROM is needed to allow for evaluation of effectiveness and impact across sites. To our knowledge there is no guideline or consensus on criteria to select HROM within a PHC setting.

## Study purposes

Our primary purpose for the Delphi consensus exercise is to derive a minimum core criteria set (CCS) of items to be used to select HROM when faced with many different measures that can be used within PHC settings. Related to this we will solicit opinions about the relative weighting of criteria groups to be used when selecting HROM.

Additional purposes for the Delphi survey are to consider core areas and domains of HROM that are important to collect in PHC and reflecting the complex interventions that are flexible and adaptable (such as Health TAPESTRY). The CCS will assist in selecting HROM but it will not provide guidance as to the core health areas of importance for PHC. Therefore, a second purpose of this study is to elicit opinions about core areas and domains and HROM that effectively capture the attributes of interest within these core areas/domains that are optimal for use within PHC settings.

## Methods/design

### General approach

**Fig. 1:**
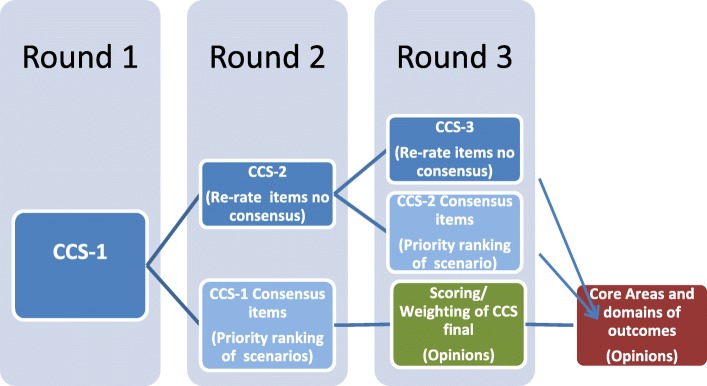
Anticipated rounds of the Delphi consensus exercise and timing of soliciting opinions on clinical scenarios and core outcomes

The stated objectives of this study require consensus on criteria for selecting HROM and opinions about areas and types of outcomes to be used in PHC settings and Health TAPESTRY. There are a number of methods used to solicit group opinion and consensus and these include nominal techniques, Delphi consensus, and consensus development conferences [34]. From these approaches we selected the Delphi consensus as the most appropriate for the purposes of this study. The global aim of the Delphi consensus exercise is to achieve consensus on a CCS for selecting HROM relevant to PHC and Health TAPESTRY. Figure 1 shows the expected rounds of the survey to achieve consensus for CCS.

Health TAPESTRY centres will also need to consider what key health areas or domains should be captured (for example physical function is a core area of health and within this there are several domains that might include activities of daily living and physical activity levels). We will solicit the opinions of the Delphi panel about core areas and domains and important HROM that they feel meet the CCS and are relevant for PHC settings and complex interventions such as Health TAPESTRY. We will not require consensus with regards to core areas and domains suggested by participants.

### Key assumptions and definitions

#### Defining HROM

The types of HROM we are considering in this Delphi consensus study are likely standardized measures of health status, disability, impairment, or handicap, as well as other clinical tests. They assess specific dimensions of a disease or a health problem (e.g., depression or frailty or fasting glucose levels). Many of these measures can be uni-dimensional (reflecting a single health construct) or multi-dimensional (several health constructs).

A number of different measures reflecting the patients’ health experiences can be used in the PHC setting and these include:

1. PROM (e.g., visual analogue scale [VAS], Short Form 36 [SF36]): PROM’s are measures that are reported or collected directly from the patient. PRO/PROM may be collected via self-administered questionnaires completed by the patient themselves or via interviews of the patients ensuring their views are adequately captured.
2. PerfO (e.g., Timed up and go test): PerfO are those that require assessment of the patient’s capacity to perform pre-specified tasks. Typical performance-based measures include the Berg Balance Scale or the 50 ft Walking Test.
3. ClinRO/ ObsRO (e.g., Edmonton Frailty Index, Functional Index Measure [FIM]): Outcomes that include clinical scales are measures where clinicians/outcome assessors evaluate the health construct of interest. This can include laboratory tests.

#### Defining primary health care

We have selected the PHC care context for our study, as it encompasses a general approach to health policy and service provision that reflect the core principles espoused by the World Health Organization (universal access, health equity, community participation, inter-sectoral approaches to health) [35] and recognizes the importance of the broad determinants of health [36]. PHC focuses on patient and provider relationship, as well as organized care. Thus, PHC embraces a wide suite of services and involves a broad range of health care providers [37, 38] that include the following: i) family physicians/general practitioners; ii) nurses, nurse practitioners; iii) rehabilitation professionals (e.g., occupational therapist); iv) physician assistants; v) nutritionists and; vi) behaviour counselors (e.g., social work).

The selection of HROM in the context of PHC can encompass a variety of outcomes reflecting domains of efficacy, effectiveness, patient engagement, patient satisfaction, and other aspects of care. Given the range of health care providers, the intended purposes of the use of the outcome may also vary. Further the selection of outcomes may need to reflect the series of complex interactions and interventions that are part of the services provided in PHC.

### Selecting core areas and domains from which to map outcomes

Core areas and domains represent the key aspects of health that the HROM attempts to capture. For example, physical function is a core area of health. Domains within the core area of physical function could include physical activity or lower extremity mobility. It is anticipated that a community adapting an intervention or putting a new intervention in place may choose different HROM that capture important core areas and domains relevant to PHC. As noted previously, our literature review identified one initiative to develop criteria for selecting outcomes in the context of clinical trials and the need for developing a core outcome set (COS) [38]. This review stemmed from the recent work from Core Outcome Measures in Effectiveness Trials (COMET) [39, 40] and OMERACT [39, 41–44] suggesting approaches to developing core areas and domains for selecting outcomes. The aim of this approach is to establish key areas of health and then select HROM to consistently assess across different research and clinical initiatives. The challenge with selecting a core set of HROM is that most initiatives are centred on a single disease and in the PHC context there are multiple health conditions often for the same patient.

**Fig. 2:**
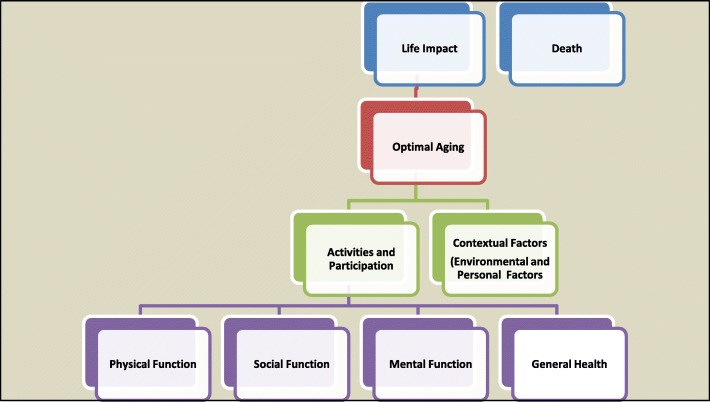
Core areas for health-related outcome measures (HROM) to be used in primary health care

We have specified a broad set of core areas (see Fig. 2). The literature suggests that the selection of HROM is related to the purpose and core area/domain of health that is of interest. At this time there is no literature specifying core areas/domain of relevance to PHC. Therefore a secondary aim of the Delphi consensus survey is to solicit opinions of the Delphi panel on the relative value of the core areas, domains and subdomains that has been preselected by Health TAPESTRY investigators. Additionally, we will solicit opinions on important HROM that would capture the aspects of health of interest and meet the core set of selection criteria established through the Delphi consensus exercise. Opinions about core areas, domains and subdomains, as well as suggested HROM will assist different Health TAPESTRY centres in selecting these measures (once the core criteria are established).

### Development of the Delphi questionnaire for CCS

#### Literature review

The investigators assembled potentially relevant criteria for selection of outcomes from a targeted literature review. We searched Pubmed using terms related to outcomes *(*i.e.*, outcome measures, Patient reported outcomes, Comparative effectiveness, Patient-centered outcomes research, Psychometrics, Measurement properties, Questionnaire, Patient Registry, Methods*). We also searched specific websites (i.e. FDA and others listed below) related to outcome measures. The citations listed below were important sources of from which our criteria was selected and these included:

1. Consensus-based Standards for the selection of health Measurement Instruments (COSMIN) checklist) [45–49]; the checklist establishes which measurement properties should be evaluated in health related patient-reported outcomes and is used to evaluate studies that attempt to establish the properties of a health related patient-reported outcomes.
2. International Society for Quality of Life Research (ISOQOL) which recently set minimum standards for patient-reported outcome measures used in patient-centered outcomes and comparative effectiveness research [1].
3. Theoretical paper discussing outcome measure assessment [50].
4. Examples of systematic reviews evaluating outcome measures [51, 52].
5. Criteria used on the GEM wiki (www.gem-measures.org) [3]
6. Issues related to harmonizing patient reported data elements in registries and health records [2, 53].
7. Reports on registries for reporting patient outcomes [26].
8. Patient Reported Outcome and Quality of Life Instruments Database (PROQOLID) (database identifies and describes clinical outcome assessments [COA’s]) to help choose appropriate instruments and facilitate access to them [54].

#### Structure of the consensus survey

**Fig. 3:**
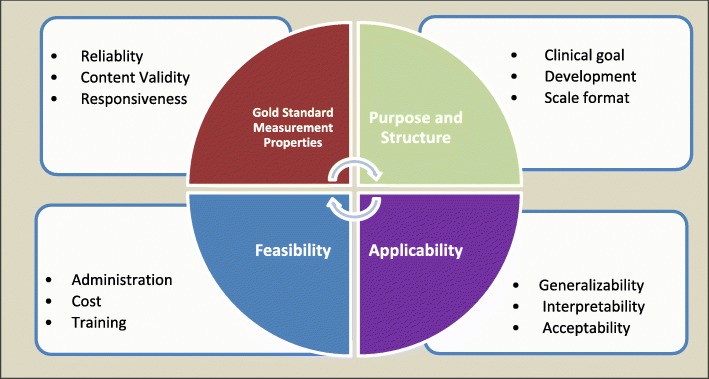
Categorization (grouping) of criteria to consider for selecting outcomes in primary health care; some examples of criteria within each categorization are shown

The criteria derived from the targeted literature search (detailed above) were grouped into four primary categories reflecting aspects of the outcome and these included: 1) gold standard measurement properties; 2) purpose and structure; 3) applicability; and 4) feasibility. Figure 3 shows some examples of subcategories of criteria within the four broad categories. Table 2 shows the criteria proposed to Delphi panel members for their consideration.

**Table 2:** Criteria found in targeted literature search and the items identified as potential criteria to be used when selecting HROM in clinical settings

Gold standard properties (*N* = 8)	Purpose, development & structure (*N* = 10)
1) Reliability 2) Content validity 3) Construct validity 4) Criterion validity 5) Cross-cultural validity 6) Responsiveness 7) Population norms 8) Diagnostic accuracy 9) Missed any criterion	1) Clinical goal 2) Specificity of the measure (disease/ generic) 3) Theoretical framework 4) Modifications since original development 5) Number of items and subscales 6) Recall period/ Time interval 7) Scoring method 8) Direction of effect 9) Literacy level 10) Translation to different languages
Feasibility (*N* = 9)	Applicability (*N* = 8)
1) Mode of administration 2) Format 3) Time to complete 4) Potential for harm 5) Need for specific skills for those that administer 6) Specialized equipment required 7) Cost 8) Accessibility 9) Harmonization potential	1) Setting and population used in validation 2) Validation of measure for use with proxy participants 3) Interpretability by patients 4) Interpretability by clinicians (and policymakers and researchers) 5) Burden to patients 6) Burden to clinicians/staff/ data collectors (volunteers) 7) Value added 8) Recognized or frequently used

From the targeted literature search a total of 35 criteria have been identified for Panel members to consider in Round 1 (see Table 2). In Round 1 panel members are asked to rate whether they view the criterion as one that could be used to select amongst different HROM and then asked to comment on their reasons. For each criterion, Delphi participants can view a short description of the conceptual basis/definitions and where possible references or sources associated with it were noted.

For criteria that has reached consensus, Delphi participants are asked additional questions in Round 2. Delphi participants are asked to comment on potential for differences in inclusion or exclusion of criterion relative to the four clinical goals/scenarios (i.e., engage, assess, screen, monitor). Prior to launch of the Delphi consensus survey pre-testing will be undertaken to ensure correct functioning of the survey online screens and for wording of the questions and notes provided to participants.

### General approach for soliciting opinions about core areas in round 3

#### Developing questions related to core areas and domains of outcomes

**Fig. 4:**
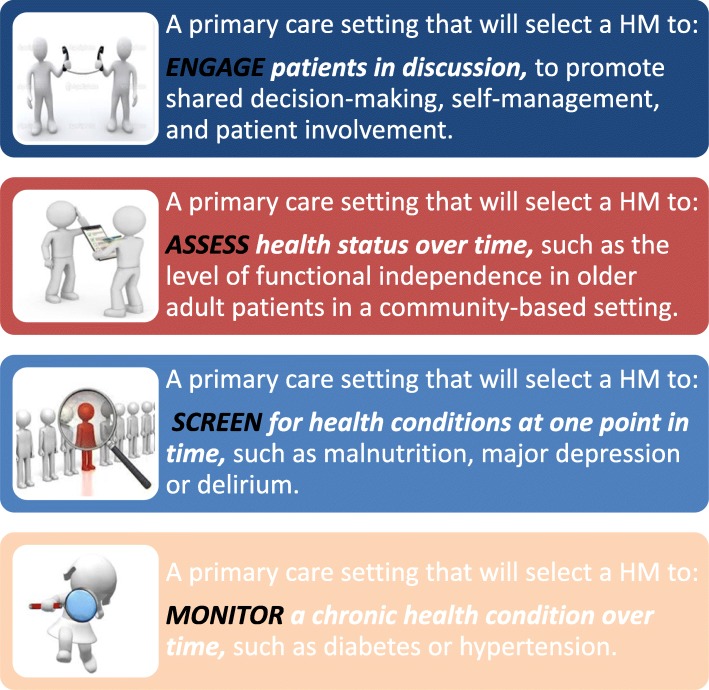
Four clinical purposes of using outcomes and these include activities that Engage, Assess, Screen, and Monitor primary health care patients

Figure 4 shows core areas and domains that the investigators selected after review of the literature of frameworks for core outcome measures [38]. The investigators believe these core areas are comprehensive and important to PHC. We will solicit opinions from the Delphi panel regarding suitable domains and subdomains to be captured consistently in primary care. We will also solicit opinions about HROM suitable for primary care settings within the suggested domains.

#### General structure of questions about the weighting of core items

These questions will be open ended and will be determined based on the results of the previous 2 rounds.

### Selection of stakeholder groups and nominated participants

Our general approach is to select individuals who have knowledge of the outcome and outcome assessment and with clinical or research experience in PHC [55]; the goal is also to have representation of different perspectives with respect to the use of HROM or the professional role of those who use the HROM [55]. For this reason we will use a purposive sampling approach. The selection of Delphi panel members should reflect the population that is intended to use the CCS or be informed by the research. In order to ensure validity of the final CCS, the panelists should reflect a diverse range of stakeholders, and likely from representing different geographic regions within Canada or internationally.

#### Identifying relevant stakeholders

There are two sets of issues to consider when selecting participants in the Delphi exercise to achieve consensus for the selection of criteria for choosing outcome measures in PHC settings and Health TAPESTRY. The first is with regards to the stakeholders and potential areas of expertise or representation from which to recruit potential panel members. We will identify key stakeholder groups and these include: i) PHC team members (all health professional disciplines of relevance) including those likely to be involved in the Health TAPESTRY across Canada (including sites in Ontario, Quebec, Saskatchewan, NFLD, and British Columbia); ii) management or administration team members in PHC clinical care sites; iii) health and health services researchers with experience in primary care HROM (including quality indicators); iv) experts in administrative issues related to collecting measures (including harmonization related issues); v) methodologists working in outcomes-related and questionnaire development research; and vi) community service organizations involved in patient care or support. Patients and volunteers will not be represented in the Delphi panel membership.

### Invitation and reminder to Delphi panel participants

All Delphi participants identified will be invited to complete each Delphi round, unless they indicate withdrawal from the study. Continuation of the Delphi participant (by linking to the survey) will be assumed as consent to participate. Reminders will be sent every 14 days following distribution of the survey. Round 1 will be closed after 12 weeks.

### Sample size for the Delphi panel

There is no set standard for sample size of a panel but it is generally agreed that the more members will increase the reliability of group judgments [34]. In has been suggested that a minimum number of panel members [56] would range from 10 to 18 panel members per area of expertise. Given the complexity of the criteria and the number of different health professionals likely with multiple areas of expertise, we will aim towards a minimum sample size of 50 participants. We will send email invitations to approximately 60 potential panel members assuming a 20% rejection rate, yielding a final sample size of 50 panel members. We are also allow for a “snowball sampling” approach, which we anticipate will increase our sample; we will encourage the pool of potential panel members to send invitations to other potential relevant participants.

#### Sampling strategy and response rate

A combined strategy will be used to select Delphi panel members. A purposive sample of panel members representing relevant professional and administrative stakeholders within PHC will be selected.

A snowball sampling approach will also be used to recruit potential panel members for the Delphi consensus exercise. We will ask the purposive sample of panelists to suggest names (and email contact) of other relevant stakeholders. Potential panel members who have experience in PHC practice or outcome measurement research will be considered.

A response of 75% is suggested for each round to achieve or maintain rigour [57] and we will monitor response rate for each round. There is the potential to introduce “withdrawal bias” if participants choose to withdraw after the first round. If Delphi panel members choose to not continue participation, we will attempt to solicit reasons for withdrawal (e.g., not enough time, too complicated, not interested) in order to assess possible threats to validity of the Delphi process [58].

### Invitation, reminders and data management of panel member information

Each participant will be allocated a random identification number and analysis of results through LimeSurvey 2.0. Demographic data (collected at the end of Round 1) will be collated and summarized as exported by LimeSurvey 2.0.

### Selection role of the Steering Committee members

Steering committee members will be selected from our team of investigators. The Steering Committee will function to summarize responses from the iterative Delphi consensus rounds. The Steering Committee will be responsible for preparing group feedback to panel respondents, and to identify any concerns with moving forward to reaching consensus. Although the intent of the Delphi consensus  exercise is to allow panel members to judge and filter the information provided, the Steering Committee may need to make some decisions to reduce items if consensus is not achieved on many criteria. This approach may be necessary to prevent risk of over burden to Delphi panelists for subsequent rounds [57]. A reduction in items will be communicated to panel members and the opportunity to respond to the feedback will be provided. This will ensure that any potential for bias is identified and rectified in subsequent rounds [57].

### Delphi consensus methods

The Delphi method has been used in a variety of research contexts to achieve both consensus [34] and dissensus [59] when soliciting stakeholder or expert opinion. The Delphi approach, is typically selected as an unbiased process to solicit opinions and reach consensus when there is no clear evidence for the solution to the problem under consideration. Stakeholder or expert (commonly referred to as the panellists, participants or respondents) consultation is required. Developed in the 1950’s and 60’s in a RAND study [57] the methodology has been key aspects that include: i) anonymity of panel members; ii) feedback is controlled and fed back to panelists; iii) survey and feedback is iterative (usually 3 successive rounds); and iv) iteration of rounds until consensus is achieved (or the law of diminishing returns sets in). The advantages of the Delphi process are that opinions of experts and other stakeholders are collected in a non-confrontational manner, free from group pressures, while group opinions are fed back to panelists. The process may help to identify aspects of the problem or solution under consideration that may have been missed or may have thought to be of limited relevance.

### Defining and achieving final consensus and timeline

For the purpose of this study, consensus is defined as general agreement of a substantial majority (75% or greater) of Delphi panelists. Re-evaluation of items would continue with iterations of Delphi rounds (a minimum of 2) or until consensus is reached on all criteria within the four groupings. If consensus cannot reached after 5 rounds, the Steering Committee will stop rounds and finalize the items within the CCS. In accordance with improved reporting standards, consensus is thus defined a priori, with specific quantitative thresholds to achieve consensus, with rounds specified as an expected 3 and up to 5, criteria for excluding and including criteria and feedback on why items were dropped or added [60]. We have specified how we will invite and select Delphi participants in a manner that is reproducible [60].

It is anticipated that we will require three rounds and expect analysis between the rounds will take up to 12 months. Round 1 commenced on January 15 2016.

#### First round

As the Delphi method involves a series of rounds in order to achieve consensus, different activities will occur at each successive round. In the first round, panel members will link to a web-based version of the Delphi questionnaire in LimeSurvey 2.0, which includes background information, instructions to the survey, and help files for specific questions. In this Round 1 Delphi panelists will be asked to rate: the appropriateness of the criterion for selecting HROM, and 2) the reason why the item would be selected or not.

We will ask Delphi panel members to suggest rephrasing, provide any rationale for their choices (each item in the survey has a comment box), and suggest missing or new items. Panel members will be given up to 12 weeks to respond. To enhance response rate, panelists will be sent a reminder notice after 2 weeks of the initial email requesting participation.

#### Second round

The responses from Round 1 will be aggregated, and analysed. The aggregated information will be fed back to panel members anonymously as part of the introductory material for the second round. Items where there is consensus to include in the CCS will be identified. Conversely, items for which there is consensus to exclude will also be identified. Finally, items for which there is a lack of consensus will be identified. Panel members will then be asked to reconsider the criteria for which consensus will not have been reached. Delphi panel members will be asked to review these findings and provide any comments.

Additionally, in the second round respondents will be asked if priority ranking of the criterion will change as a consequence of the clinical goal (see Fig. 1). Figure 4 identifies four clinical goals (adapted from Greenhalgh [28, 29]) that are related to the HROM being used. This will be asked for items for which there is consensus that the criteria should be included. Note that consensus is not necessary in these responses to priority ranking. Our aim is to solicit opinions with regards to ranking as a function of the clinical goal of the HROM. We will ask Delphi panel members to suggest rephrasing, provide any rationale for their choices (each item in the survey has a comment box), and suggest missing or new items.

#### Third round (or greater)

Criteria that remain indeterminate from Round 2 will be brought back into the third round or for continued series of iterations until consensus is achieved (see Fig. 1). Panel members will re-evaluate criteria for which there is insufficient consensus for inclusion/exclusion. Conversely, a third round will be omitted if consensus (on all remaining items) is reached following the second round. We will ask Delphi panel members to suggest rephrasing, provide any rationale for their choices (each item in the survey had a comment box), and suggest missing or new items.

We will add additional questions regarding the relative weighting of the four groupings of items for a final CCS for which consensus has been reached. These questions will be developed as the CCS emerges following the Delphi rounds. The aim of these questions is to solicit opinions only and not achieve consensus.

Finally, we will solicit opinions about the core areas and domains and outcomes that meet the CCS final criteria within these domains.

## Discussion

There are several important gaps in the literature with respect to how to select criteria to choose HROM and in which contexts. We consider several of these gaps here.

### Previous attempts to establish criteria for selecting HROM and their limitations

There have been several attempts to establish criteria for selecting HROM. If the focus of the outcome assessment is on matters of importance to patients, then it is clear that measures evaluating health related quality of life are important to consider. The ISOQOL has provided one such effort with the goal to establish minimum standards for the design and selection of PROM suitable for comparative effectiveness reviews and for patient centered outcomes research [1]. They assembled potential criteria from a targeted literature review identifying existing guidance (*n* = 28 guidance documents) on the selection of PROM in patient-centred outcomes research, followed by an online survey of their membership. Their recommended criteria focused on a succinct list (*n* = 7) of measurement properties (i.e., reliability, validity, etc.) and practical attributes (i.e., interpretability, acceptable burden, etc.). Although an important initial step, these criteria were developed with consideration of PROM and multidimensional instruments reflecting health related quality of life; they did not take into account the specific health setting or clinical context for use (i.e., screening, monitoring, etc.).

A second attempt to establish criteria for selecting HROM did take into account the PHC context; the criteria for selecting outcomes was considered with respect to use of the electronic health record to record PROM specifically measuring health behaviours and psychosocial concerns [2, 3]. In the inaugural phases of this initiative, content experts (i.e., expertise in physical activity, goal setting, patient literacy, anxiety and depression, etc.) were instructed to consider a set of proposed scientific and practical criteria for recommending outcomes to capture attributes of health promotion and disease prevention. From these a core set of criteria (*n* = 12) that were grouped according to “scientific” attributes and “practical considerations” were proposed. A grid-enabled measures (GEM) portal on a wiki site provided a repository of recommended psychosocial outcome instruments and allowed other stakeholders (researchers, practitioners, administrators) to continue to make suggestions and recommendations on the use of these measures in the context of these criteria. Although the focus of these criteria was targeting only selection of PROM for behavioural and psychosocial issues and considering the electronic health record in PHC, real world contexts were considered and reflected in the “practical considerations” components of the criteria.

A third set of core criteria were developed by the Core Outcome Measurement Instrument Selection (COMIS) [4, 20] in cooperation with the COMET Initiative and the COSMIN. The criteria was derived using a Delphi consensus exercise (that included both research and clinical experts) with the intended goal of developing a guideline on how to select measurement instruments included in a Core Outcome Set (COS). A COS is an agreed minimum set of outcomes that should be measured and reported in all clinical trials of a specific disease or trial population. The definition of an outcome considered in this research is one that conceptualizes outcome as the “what” is being measured and defined as a “construct or domain”. It follows then that an outcome measurement instrument captures these constructs. Prinsen et al., (2016) [4] did not restrict their criteria to any type of instrument (e.g. included PROM but is all inclusive) and included experts with both clinical and research experience. However, the focus during development of these criteria was to develop a minimum set of outcomes for use in clinical trials for a disease or patient population rather than a health service or setting.

The fourth initiative to identify criteria to select health related measures generally and HROM was developed by the Institute of Medicine (IOM) which examined core measures used within the American health care system and considered some criteria for core measures and core sets. The IOM committee aimed to decrease inconsistent and duplicative efforts to collect HROM when assessing system level quality and established criteria for core measures [5, 6]. For individual patient outcomes these included: consideration of the measures’ importance to health, strength of linkage to progress, understandability of the measure, technical integrity, potential for broader system impact, and utility at multiple levels. Additional consideration was given to system related issues and these included: systemic reach, outcomes oriented, person meaningful, parsimonious, representative, and utility at multiple levels [6]. The IOM effectively considered both individual and system level categories that could be applied to PHC and likely capturing complex interventions.

These previous attempts to establish criteria to select PROM/HROM demonstrate the growing interest in establishing criteria and also justification for selecting outcomes. These previous attempts provide important context for what criteria have been considered and how these were developed. There are areas of overlap across these four different sets of recommended criteria which reflect a convergence of key attributes that may be grouped into those reflecting measurement properties and practical aspects. However, there are differences in items within these broad categories across criteria sets, which may in part reflect the specific purposes of each of their respective development processes. The influence of the health care setting, particularly PHC and the complex nature of this environment, is not clear in the development of these criteria and would merit further exploration.

Although helpful, these previous attempts at establishing a CCS may not be applicable and feasible for application in PHC settings where patients with multiple health conditions and complex interventions are the typical and the constraints of providing health services differ from those in research studies. We have proposed a methodology to conduct a Delphi and constructed an online survey which we pilot tested for comprehension and pre-tested to ensure adequate functioning of the survey set up. Although we did not undertake cognitive debriefing but believe the pre-testing will take this into account.

### Understanding the context of primary care and primary health care

There is some inconsistency in understanding differences between primary care and PHC [35] and the differences are typically around the types of services provided. For the purposes of this paper we refer to primary care where there are interactions and services with clinicians (usually general practitioners) as first line of service. [35]. In contrast PHC is broader in scope and encompasses a general approach to health policy and service provision (i.e. core principles that include universal access, health equity, community participation, inter-sectoral approaches to health promoted by the World Health Organization) [35]. Effective PHC is community-based, promotes healthy lifestyles to prevent illnesses, considers ongoing care for chronic conditions and acknowledges the importance of the broad determinants of health [36]. There is evidence to show that PHC helps prevent morbidity and mortality and is associated with more equitable distribution of health in the populations [21].

PHC also reflects the first contact with the health care system and coordination and integration thereby ensuring access to care (diverse services and health providers) [36, 37] that include the following: i) family physicians/general practitioners; ii) nurses, nurse practitioners; iii) rehabilitation professionals (e.g., occupational therapist); iv) physician assistants; v) nutritionists and; vi) behaviour counselors (e.g., social work).

Understanding the role of PHC in the health of a population, the rationale for selecting HROM is compelling. The selection of HROM in the context of PHC can encompass a variety of outcomes reflecting domains of efficacy, effectiveness, patient engagement, patient satisfaction, and other aspects of care. Given the range of health care providers, the intended purposes of the use of the outcome may also vary. Further the selection of outcomes may need to reflect the series of complex interactions and interventions that are part of the services provided in PHC.

### Availability of multiple HROM

We anticipate that there are many HROM that possess good aspects and this presents a new dilemma; that is, selecting from several HROM attempting to capture the same health attribute of interest that meet the established criteria for selection. The stakeholder is faced with having a rationale for their choice. One potential strategy to address this issue is to consider “weighting” some items as more important over others; soliciting opinions from the Delphi panel on the final set of criteria for which consensus is achieved may provide some practical direction for implementation.

Soliciting opinions about important core domains for outcomes may also assist stakeholders in making choices amongst different HROM that meet the CCS. The conceptual models that underpin some complex or multi-dimensional HROM may need to be compared to core areas deemed appropriate or ideal for the PHC context. As noted previously, primary care patients reflect heterogeneous population with a variety of health conditions. There is limited discussion in the literature about the core areas and subdomains that ideally should be captured. Opinions from panel members will be collated and reflected back in the guidance document of for implementation of the CCS.

Because of these gaps, we developed this protocol to undertake a Delphi consensus online survey to establish criteria for a broader array of types of outcomes (i.e., HROM) specific to the context of PHC settings. A Delphi consensus exercise is a preferred method when consensus is lacking and in this case the literature suggests some lack of overlap in possible criteria. The advantages of the Delphi which include anonymity, provision of controlled feedback, and avoidance of face to face meetings will minimize potential biases due to personalities and other types of influences. Our selection of sample members will not be random and is influenced by current members of different Health TAPESTRY affiliates; the sample therefore may reflect these perspectives and not be truly representative of all primary health care contexts. The findings from this Delphi study will address a gap for establishing consensus on criteria for selecting HROM for use across PHC settings and complex interventions.

## Abbreviations

CCS: Core criteria set
ClinRO: Clinician reported outcome
COA: Clinical Outcome Assessment
COS: Core outcome set
FIM: Functional Index Measure
HROM: Health related outcome measures
ObsRO: Observer reported outcome
PerfO: Performance outcome
PHC: Primary health care
PRO: Patient reported outcome
PROM: Patient reported outcome measure
PROQOLID: Patient Reported Outcome and Quality of Life Instruments Database
SF36: Short Form 36
VAS: Visual analogue scale
